# The Role of Minimally Invasive Surgery in Mitral Valve Repair: Through the Female Gaze

**DOI:** 10.3390/jcm14186349

**Published:** 2025-09-09

**Authors:** Minji Ho, Edouard Long, Paolo Bosco

**Affiliations:** 1 Faculty of Life Sciences and Medicine, King’s College London, London SE1 1UL, UK; 2 Department of Cardiac Surgery, St. Thomas’ Hospital, London SE1 7EH, UK

**Keywords:** mitral regurgitation, mitral valve repair, cardiac surgery, minimally invasive cardiac surgery, female sex

## Abstract

**Purpose of review:** Numerous studies have investigated sex-specific differences in mitral regurgitation (MR) and mitral valve (MV) surgery. However, less is known about the possibility of sex-related outcomes in minimally invasive cardiac surgery, particularly in the case of minimally invasive MV repair. This review seeks to highlight existing evidence and identify gaps in the literature. **Findings:** Female patients with MR tend to present later, more symptomatically, and more comorbidly. This sex bias extends to pre-operative investigation, with females underrepresented in multidisciplinary evaluation and having longer intervals to surgery, often only receiving investigations as inpatients, and suffering from excess post-operative mortality compared to male patients. Very few studies directly investigate how these factors are related to female patients who undergo minimally invasive MV surgery, and fewer still interrogate sex-specific outcomes in minimally invasive MV repair. **Summary**: As cardiac surgery continues to evolve, it is of utmost importance to fully characterise the importance of sex as a factor in patient care for MR, and further research is necessary to reconcile its role in the advancement of minimally invasive MV surgery.

## 1. Introduction

Mitral regurgitation (MR) is amongst the commonest valvular abnormalities worldwide [1]. The epidemiology of MR is well documented and of significant relevance, particularly as its prevalence increases with age, with 1% of patients over the age of 55 suffering from mitral valve disease [2]. In spite of inconsistent evidence on the potentially imbalanced prevalence of MR between males and females [3], females are less likely to receive operative treatment for mitral valve prolapse than males [4] and may be more likely to suffer post-operative mortality, regardless of the type of surgical access [5]. Surgical access can include median sternotomy, also known as open mitral valve surgery, or minimally invasive techniques, including right minithoracotomy, hemisternotomy, transapical, endoscopic, or even robotic approaches [6,7]. While sex-specific studies on MR are on the rise, only a subset of these focus on mitral valve repair (MVr) via minimally invasive mitral valve surgery (MIMVS).

## 2. Classification of Mitral Regurgitation

MR can be classified according to its pathophysiological mechanism as primary or secondary. Primary mitral regurgitation (PMR) involves intrinsic valvular lesions to the mitral valve apparatus itself, while secondary mitral regurgitation (SMR) involves regurgitation predominantly caused by extra-valvular pathology, such as ventricular or atrial remodelling [8].

These valvular deformations can be further subclassified, as per Carpentier’s classification, into Type I, normal leaflet motion with annular dilatation, Type II, leaflet prolapse and/or flail, and Type III, leaflet restriction in systole and diastole (IIIa) or systole only (IIIb) [9]. Proper classification helps to guide the operator to the most appropriate intervention strategy for repair or, if necessary, replacement, based on the distinct morphology of the underlying lesion [10]. The pathophysiology of MR is not without its sex differences either—female patients may exhibit intrinsic variations in extracellular matrix remodelling that predisposes them to developing degenerative bileaflet prolapse, as well as differences in the extent of valvular calcification and an increased prevalence of rheumatic heart disease [11,12]. The literature suggests that sex discrepancies may also exist anatomically, with post-mortem studies showing lower annular elasticity and larger indexed circumference in female patients, and these morphological changes can have carry-on effects when planning and performing therapeutic interventions [13]. These differences may also be present in the prevalence of leaflet morphology and classification, with increased rates of myxomatous degeneration, particularly with anterior and bileaflet prolapse, in females [13]. This is of particular concern, as evidence suggests that the long-term surgical durability and freedom from reoperation is closely associated with the complexity of valvular disease, with posterior leaflet repair demonstrating superior outcomes to anterior or bileaflet repair [14]. As such, it is evident that sex disparities are present even within the prevalence of mitral regurgitation subtypes, which can have subsequent consequences on the presentation and outcomes of female versus male patient groups.

## 3. Sex-Specific Presentation

The literature has demonstrated that female patients, compared to males, are diagnosed at older ages and, as reported by Waldron et al., are less likely to be discussed in multidisciplinary evaluations (odds ratio [OR], female vs. male: 0.27, 95% confidence interval [CI]: 0.15–0.47, *p* < 0.001) [15], despite presenting with more comorbidities [4]. This is particularly concerning in light of advancing techniques in both open and minimally invasive cardiac surgery, particularly in the case of complex degenerative valve disease and suitability for minimally invasive access, both of which may require more extensive imaging and input from colleagues in allied specialties. Beyond the evaluation of such cases in-house, there have also been recent developments in international valvular heart disease guidelines in both Europe and America suggesting that patients with severe MR be referred to specialised centres with specific expertise in MVr [16,17]. As such, the lower propensity of females being referred to multidisciplinary evaluation may have debilitating effects on their candidacy for MVr and MIMVS.

The more co-morbid presentations of female patients, including higher rates of congestive heart failure and atrial fibrillation (AF), make females more likely to require concomitant procedures involving the tricuspid valve and/or surgical AF ablation, as their valvular disease tends to be more advanced at the time of intervention, and many are only being investigated once in the inpatient setting [4,15]. Morphologically, females present more commonly with mitral valve stenosis as an indication for intervention than males [4], although this was found to be the case in mitral valve replacement (MVR) specifically and not necessarily in MVr, by Vassileva et al. This study excluded patients with an extensive cardiac history in its patient population, suggesting that the high prevalence of heart failure in its female subgroup, both undergoing repair and replacement, is likely related to disease affecting the mitral valve rather than other causes [4].

Male patients have also been shown to be more likely to present with smoking history, chronic obstructive pulmonary disease, renal insufficiency, previous myocardial infarction, history of percutaneous coronary intervention, morbid obesity, and alcohol abuse [18]. However, at baseline, males have consistently been shown to present with lower NYHA in PMR, with mixed evidence in SMR [19]. Interestingly, some research has also been conducted to investigate the association of obesity (body mass index (BMI) > 30) with outcomes after mitral valve surgery. One study found that over half of patients with BMI > 30 and ≥40 were female, which was associated with greater risk of inpatient mortality, although this risk was insignificant with adjustment for other variables [20]. Morbid obesity (BMI > 40) was also demonstrated to markedly increase hospital stay and mortality, as well as failure to discharge back to the original home setting [20].

There also exists a difference in presentation between PMR and SMR; while females tend to present with more severe progression and comorbidities in PMR, they constitute a smaller proportion of SMR patients, with the exception of atrial SMR, with an SMR study reporting only 33% females in the study population and few significant sex differences in initial presentation [21].

## 4. Sex-Specific Assessment and Intervention

Sex-specific differences exist not only in the presentation of mitral disease but also in the assessment and strategy for intervention. It has been well-documented that female patients who present with valvular disease undergo delayed surgical correction, despite presenting with more severe echocardiography findings [22]. Avierinos et al. found that females were significantly more likely to be referred for intervention for symptomatic management (72% vs. 57%) but that only 4% of women, compared to 11% of men, were operated on the bases of left ventricular (LV) dilatation alone, a Class I indication for intervention in international guidelines [10,23]. This finding was corroborated by studies showing similar proportions, such as one by Bernard et al., which stratified triggers for surgery in both symptomatic and asymptomatic patients, of which the latter included LV end systolic diameter (LVESD) ≥ 40 mm and showed that 9.9% of males fulfilled this criteria while only 4.1% of females did [24].

These disparities exist in other cardiac investigations as well; research into cardiac magnetic resonance imaging demonstrated lower left and right ventricular volumes in women than men, even when indexed to body surface area (BSA) and age [25]. This sex imbalance was also found when examining the relationship between cardiac magnetic resonance parameters and echocardiographic parameters, with female patients presenting significantly lower indexed LV end-diastolic volume thresholds suggestive of abnormal LV remodelling compared to male patients [25]. The existence of such a stark difference in trends between sexes can have detrimental downstream effects, particularly as imaging parameters are frequently used as cut-offs for surgical candidacy. In fact, the previously mentioned study by Altes et al. found that women were often classified as having “moderate-to-severe” MR, as opposed to severe MR, due to persistently lower echocardiographic measurements of MR severity [25], a finding which was corroborated by Enrique-Sarano et al. in asymptomatic patients [26].

Surgical uptake was similar in males and females presenting with New York Heart Association (NYHA) Class III-IV symptoms; however, in patients who presented either asymptomatically or with minimal symptoms, surgical uptake was much lower and significantly more delayed in females, in spite of larger cavity dimensions, higher pulmonary pressures [27], and LVESD exceeding guideline thresholds [22]. This group of females also had a higher calculated EuroSCORE II than their male counterparts, suggesting more advanced disease progression at presentation. Females are also overrepresented in non-elective operation status, in line with investigative delays and more advanced valve disease at presentation [28]. These sex-specific differences in investigation and assessment may serve as compounding factors that influence subsequent sex disparities in post-operative endpoints.

At the time of operative intervention, Chan et al. found that female patients had more extensive mitral leaflet prolapse and calcification than their male counterparts, both in the anterior and posterior leaflets [29]. The female mitral valve apparatus is also suggested to suffer from more annular calcification [11], which may play a role in females being substantially less likely to undergo MVr [4,30], which can have knock-on effects in post-operative outcomes. In the aforementioned study, both sexes were more likely to undergo valve replacement than repair [30], despite MVr being preferred to replacement in primary MR [11]. The difference in MVr rates between sexes was reflected in a study by Berretta et al., which demonstrated female sex was an independent predictor of replacement over repair in MIMVS-specific registers [31]. There is inconsistent data on the prevalence of certain surgical techniques, such as neochord placement in the papillary muscles, across the two sexes, with some literature suggesting there is no significant difference [27], while other literature suggests female patients undergo more annuloplasty-only repair [32].

Of particular interest in the development of modern cardiac surgery is the study of sex differences in minimally invasive surgery, particularly at the crossroads of sex imbalance in valvular disease, such as in the case of MR, and how these factors can extend to MV interventions. There is a recognised gap in the literature concerning the emerging role of minimally invasive mitral valve repair (MIMVr) [33]. Dębski et al. propensity matched a cohort of men and women undergoing mitral surgery and determined both sexes had good candidacy for either surgical approach yet found that female patients were significantly underrepresented in the MIMVS group, despite being clinically similar to the male patients pre-operatively [34]. In fact, the data showed that male patients had significantly higher propensity scores or were more likely to receive MIMVS, regardless of which approach they ultimately received. This is consistent with findings from research into other minimally invasive approaches such as robotic-assisted MVr, which found that patients undergoing robotic MVr, which is associated with less pacemaker insertion and shorter hospital length of stay, were more likely to be male [35].

Interestingly, differences in mitral valve pathology between MIMVS and median sternotomy may also be indirectly related to sex-specific differences. Malik et al. reported more posterior mitral leaflet prolapse, a pathology known to be more common in males [11], in the MIMVS group [30], while anterior and bileaflet flails, which are more common in females [10], were more common in the median sternotomy groups [30]. This study was one of few that reported a higher rate of MVr than MVR in its patient population, suggesting a stronger need to investigate the role of sex-specific differences in MVr through MIMVS. A similar study conducted on exclusively MIMVS found a 99% (*n* = 198) success rate for repair, with two failed cases due to severe calcification and post-operative systolic anterior motion of the mitral leaflet [36]. This literature suggests a strong case for the progressing use of MIMVS for repair over replacement.

## 5. Sex-Specific Outcomes in MVr and MIMVS

The literature has demonstrated a strong interest in the comparison of median sternotomy versus minimally invasive cardiac surgery, particularly in the case of valve replacements. However, a consensus has yet to be reached on whether MIMVS is decidedly superior to conventional open access. Within this interest, a smaller niche focuses on sex-specific outcomes for MVr in particular.

Observational analyses have suggested that females suffer significantly higher postoperative mortality than males, regardless of surgical access and procedure type, with the exception of isolated aortic valve replacement [4,5,15,18,19,30]. Concerningly, MVr has been shown to restore male patients to their normal life expectancy but not female patients [4]. It is clear that there exists a sizeable gap in outcomes between males and females, but the degree to which this is affected by surgical access remains under-studied. In spite of this evidence, a 35-year study by Lazam et al., comparing MVr and MVR, demonstrated that repair is superior to replacement regardless of age and sex and, interestingly, regardless of the calendar year of operation [32]. This improvement in long-term survival was demonstrated to increase over time, even in the presence of other coronary risk factors and across all types of valvular prolapse classification [37]. The study, which followed up participants 20 years post-operatively, demonstrated freedom from grade 3+ MR in 88% (95% CI 86–90%) post-repair across the total population. Besides mortality and efficacy of intervention, females also suffer in other post-operative outcomes including length of stay, particularly in the case of repair, as demonstrated by our group [38]. This study by Long et al. also demonstrated that urgent operation status was a predictor of mortality in female patients but not in their male counterparts [39].

Evidence also exists in support of MIMVS achieving superior outcomes to median sternotomy, particularly when it comes to ventilation time, intensive care unit (ICU) stay, intra-operative and post-operative blood requirement, and bleeding [32]. An analysis by Taghizadeh-Waghefi et al. showed no inpatient mortality after MIMVS post-operatively compared to 3.5% in the median sternotomy group (*p* = 0.02) but also no significant difference in major post-operative outcomes, including stroke, myocardial infarction, respiratory failure, pacemaker insertion, new onset AF, and occlusion of the circumflex artery. This study boasted a “single incision-direct vision” approach that did not necessitate use of an endoscopic camera to guide operative technique and was therefore less invasive than the traditional median sternotomy; however, it acknowledged that the actual intervention taken to the mitral valve was comparable to open surgery, with full cardiopulmonary bypass (CPB) established. Interestingly, while the data showed longer CPB and cross-clamp times in the MIMVS group compared to the median sternotomy group (mean MIMVS CPB time < 90 min, cross clamp time < 65 min), this did not translate to a longer total operation time. This serves as a notable contrast to the existing literature from Germany demonstrating mean CPB times of over 180 min when investigating different types of MIMVS incision and access [40], which may serve to demonstrate discrepancies in the various minimally invasive techniques and the importance of referring to expert valve centres.

Other research highlights that, while there may be no difference in post-operative mortality between MIMVS and median sternotomy subgroups in females undergoing mitral valve surgery (MVS), there may be a significant difference in post-operative mortality between male and female patients in both the MIMVS and median sternotomy sub-groups [5]. As demonstrated by Moscarelli et al., the female MIMVS group had shorter ventilation time (12.9 h (SD: 51.1 h) vs. 15.2 h (SD: 72.3 h), *p* = 0.014), although other variables were not significantly different [5]. This study was able to adjust for baseline covariates and demonstrate that, while MIMVS is not inferior to median sternotomy, both in females and in the total study population, MIMVS did not mitigate the inherent risk of inpatient mortality and post-operative complications in females. It must, however, be noted that the female cohort comprised a majority of repairs, although also included a substantial number of replacements in its dataset (MVr: 1269 (76%), MVR 401 (24%)).

Long-term survival appears to show less of a difference between sexes, as summarised by Johnston et al., with no significant difference at 5 years in a collection of studies from the Netherlands [41], Canada [27], and the United States [18]. However, it is difficult to determine if this finding is applicable to MIMVS, particularly as many MIMVS versus median sternotomy studies use propensity score matching. When controlling for confounding factors, using sex during cohort matching may overlook the possibility of sex-specific outcomes, as well as increase potential bias when selecting candidacy for MIMVS before intervention, particularly in the case of MVr, which has been shown to be underrepresented in female patients with MR. Such an example can be seen in Paparella et al., where, in pre-propensity score matching, sex shows a significant *p* value (<0.001), which is eliminated post-matching [37].

## 6. Application of Transcatheter Therapies

The advent of minimally invasive therapies has also generated new advances in transcatheter techniques, as well as surgical. In the context of transcatheter edge-to-edge repair (TEER), a registry of 1142 patients was analysed by Biasco et al. and demonstrated no significant sex differences in outcomes between males and females, although the study population consisted of just over 60% males [42]. This disparity has been reflected in other transcatheter research, highlighted in a review by El-Andari et al., in which all but two studies from 2006–2018 demonstrated a sex imbalance in study populations [43]. This analysis also suggested that female patients do not suffer the same sex-associated inferiority in outcomes as their surgical counterparts. Yet, despite seemingly positive outcomes in TEER repair, the literature continues to show that females are underrepresented in TEER for MR, with large-scale American trials such as Cardiovascular Outcomes Assessment of the MitraClip Percutaneous Therapy for Heart Failure Patients with Functional Mitral Regurgitation (COAPT) consisting of only 36% females and Endovascular Valve Edge-to-Edge Repair Study (EVEREST-II) only 36.2%, [33,35]. This is consistent across European trials as well, with Percutaneous Repair with the MitraClip Device for Severe Functional/Secondary Mitral Regurgitation (MITRA-FR) including only 25.3% female patients and the EuroSMR registry, which focused on SMR, including 36%, although this may be mitigated by the inherently lower prevalence of SMR in females [44,45]. Interestingly, while SMR appears to predominantly affect males, atrial SMR as a subgroup is suggested to disproportionately impact female patients, which may, in turn, cause adverse outcomes as studies demonstrate worse outcomes in atrial SMR compared to PMR after TEER [13].

Interestingly, a study further explored sex differences in transcatheter mitral valve replacement (TMVR) selection in detail, showing that only 36.7% of eligible candidates were female but, more critically, that more females went on to receive high-risk surgery over “bailout” TEER, unlike their male counterparts [46]. This may be explained by a further analysis, which showed that most high-risk surgery referrals were for cases of PMR with increased mitral annular calcification, which, as previously described, is more prevalent in female patients [46].

Disease morphology has also been demonstrated to show consistent outcomes between surgical and transcatheter approaches. MR classification can have direct impacts on subsequent management, as SMR patients often have worse outcomes both intra-operatively and post-operatively, including lower rates of repair and higher 30-day and one-year mortality [47]. These outcomes persist with transcatheter interventions, as demonstrated by the CUTTING-EDGE registry, which investigated patients undergoing MV surgery after TEER and found high mortality both 1 and 3 years post-intervention [47].

## 7. The Sex Gap

Very few studies explicitly interrogate sex-specific differences in MIMVr, despite rapidly advancing interest and progress in the literature documenting sex-specific differences in MR and MVS as well as in MIMVS versus median sternotomy. It is also challenging to determine where a potential knock-on effect lies, as the literature demonstrates a variety of potential causal factors in sex-specific outcomes from MVS, including delayed and advanced presentation and lack of multidisciplinary evaluation, as opposed to inherent sex differences in efficacy of MVr.

There exists a notable gap in the literature seeking to reconcile the uncertainty between the impacts of sex on outcomes in MVr and the role of MIMVS in repair over replacement. Research strongly suggests that, regardless of the underlying reason, female patients receive unequal treatment to male patients and suffer worse outcomes in MVr. However, as greater attention is paid to female-specific outcomes, advances in reconciling research are reassuring, such as in the case of the Randomized researcH in womEn all comers wIth Aortic stenosis (RHEIA) trial, which directly compared the efficacy of transcatheter valve implantation versus conventional surgery in female patients with aortic stenosis [39]. The findings of this randomised controlled trial, which support minimally invasive approaches, highlight the increasing need for better understanding of female outcomes and draw attention to the need for a comparable trial in other valvular diseases, particularly those that disproportionately affect women, such as MR. Such a trial could also highlight sex-specific benefits to certain surgical approaches, especially given the diversity of techniques in MIMVS and MIMVr.

It is not only the treatment of MR that shows potential sex-specific differences. It is evident that investigation pathways also display disproportionately negative outcomes for female patients, particularly with the timeliness and multidisciplinary approach to deciding suitability for surgical intervention. Current European and American guidelines do not reference sex-specific recommendations in the investigation of MR [10,23] despite the literature documenting significant differences between male and female echocardiography measurements, including increased left atrium (LA) stiffness, irrespective of age and other imaging assessment [40]. Prior research on LVESD has demonstrated a clear cutoff point at which hazard ratios for mortality increased in women (3.6 cm), a relationship which does not significantly exist in male patients [41]. This is a notable discrepancy that is entirely unaccounted for and may be implicated in the numerous studies reporting sex-specific differences in the assessment, management, treatment, and outcomes of mitral repair cited in this review. As echocardiography evaluation forms the cornerstone of MR investigation and management, it is crucial that guidelines are adapted appropriately to account for sex-linked differences. The current guideline of an LVESD of 4 cm exceeds the 3.6 cm point at which mortality increases for female patients, highlighting the need for further investigation and validation of the possibility of sex-specific differences in the investigation and referral of MR for intervention to address potential gender biases more systematically before the patient reaches the operating table. Numerous studies have corroborated findings of LVESD discrepancies between male and female patients presenting for MV surgery, including after propensity matching. One such study showed that, even after covariate adjusting, female sex remained a risk factor for recurrent MR after intervention [48]. Furthermore, some research into post-operative echocardiographic parameters has suggested that female patients may undergo less positive LV remodelling after intervention, even when LV measurements are indexed to BSA and other factors are adjusted for [29].

While reviews and analyses have amalgamated the available findings on sex-specific differences in MIMVr, the lack of deliberately structured and standardized data limits its applications. As sex is frequently treated as a confounding factor to be balanced on propensity matching, purposeful study design is essential to building a more robust database on sex-specific differences to ensure that other variables are taken into account and matched accordingly to isolate sex as a risk factor. Additionally, given the often more limited caseload of MIMVS and MIMVr, it would be beneficial to derive a standardised protocol and comparison between techniques to better evaluate how sex plays a role in deciding candidacy and whether the selection and procedure are influenced by sex-related discrepancies, perhaps on a prospective basis, to improve the validity and replication of existing retrospective findings.

## 8. Conclusions

While there has been development in the recognition of sex-specific differences in the diagnosis and treatment of MR, their implications have not been well-translated to the guidelines used in investigations and surgical referrals. Numerous studies have investigated the presentation and comorbidity burden of MR in females, including quantitative differences in investigations, yet global cut-offs still use absolute values for LVESD, despite increasing evidence showing that female patients suffer adverse outcomes at lower thresholds. In the present era furthering precision medicine and minimally invasive approaches, there is notable capacity for more tailored assessment of female patients, which could be supported by more robust evidence derived from direct comparison of patient sex in multiple modalities of treatment, including MIMVS. The literature strongly suggests that female patients suffer disproportionately with their access to and outcomes from MVS, both in the apparent differences in fundamental presentation with MR and in their candidacy for MVr and MIMVS procedures. While there has been some research into sex-specific differences in these areas, the data is limited, particularly for studies which account for significant heterogeneity within sexes and, further yet, investigate them in a randomised controlled trial. As cardiac surgery evolves, both in social equity and innovation, it is essential that precision medicine adapts to integrate sex as an important factor in assessing, managing, and treating cardiovascular diseases to bridge the gap between patient groups.

## Abbreviations

The following abbreviations are used in this manuscript:

MR	Mitral regurgitation
MVr	Mitral valve repair
MIMVS	Minimally invasive mitral valve surgery
PMR	Primary mitral regurgitation
SMR	Secondary mitral regurgitation
NYHA	New York Heart Association
BMI	Body mass index
LV	Left ventricle
LVESD	Left ventricular end systolic diameter
BSA	Body surface area
MIMVr	Minimally invasive mitral valve repair
ICU	Intensive care unit
CPB	Cardiopulmonary bypass
TEER	Transcatheter edge-to-edge- repair
LA	Left atrium
MVS	Mitral valve surgery
